# Integrated Analysis of Transcriptome and Secretome From Umbilical Cord Mesenchymal Stromal Cells Reveal New Mechanisms for the Modulation of Inflammation and Immune Activation

**DOI:** 10.3389/fimmu.2020.575488

**Published:** 2020-09-30

**Authors:** Mónica Cruz-Barrera, Nathalia Flórez-Zapata, Nicolás Lemus-Diaz, Carlos Medina, Cristian-Camilo Galindo, Lorena-Xiomara González-Acero, Luz Correa, Bernardo Camacho, Jens Gruber, Gustavo Salguero

**Affiliations:** ^1^Advanced Therapies Unit, Instituto Distrital de Ciencia Biotecnología e Innovación en Salud – IDCBIS, Bogotá, Colombia; ^2^Universidad EIA, Envigado, Colombia; ^3^Junior Research Group Medical RNA Biology, German Primate Center, Leibniz Institute for Primate Research, Göttingen, Germany

**Keywords:** UC-MSC, inflammation, RNA-seq analysis, MSC-immune modulation, IL-2Ra

## Abstract

Mesenchymal stromal cells (MSC) have been used in over 800 clinical trials with encouraging results in the field of transplant medicine and chronic inflammatory diseases. Today, Umbilical Cord (UC)-derived MSC are the second leading source used for clinical purposes, mainly due to its easy access and superior immune modulatory effects. Although the underlying molecular mechanisms of immune suppressive activities have not been fully understood, research over the last decade strongly suggests that MSC-mediated benefits are closely related to activation of secretome networks. Nevertheless, recent findings also point to cytokine-independent mechanisms as key players of MSC-mediated immune modulation. Here, we set up a robust *in vitro* immune assay using phytohemagglutinin- or anti-CD3/CD28-treated human peripheral blood mononuclear cells in cell-to-cell interaction or in cell-contact independent format with UC-MSC and conducted integrated transcriptome and secretome analyses to dissect molecular pathways driving UC-MSC-mediated immune modulation. Under inflammatory stimuli, multiparametric analyses of the secretome led us to identify cytokine/chemokine expression patterns associated with the induction of MSC-reprogrammed macrophages and T cell subsets ultimately leading to immune suppression. UC-MSC transcriptome analysis under inflammatory challenge allowed the identification of 47 differentially expressed genes, including chemokines, anti- and pro-inflammatory cytokines and adhesion molecules found also in UC-MSC-immunosupressive secretomes, including the novel candidate soluble IL-2R. This study enabled us to track functionally activated UC-MSC during immune suppression and opened an opportunity to explore new pathways involved in immunity control by UC-MSC. We propose that identified immunomodulatory molecules and pathways could potentially be translated into clinical settings in order to improve UC-MSC-therapy quality and efficacy.

## Introduction

Mesenchymal stromal cells (MSC) are instrumental in modulating immune responses in the context of inflammation. Based on cumulative evidence over the last decade, MSC have emerged as an attractive platform to develop cell-based immune therapies by exploiting their molecular machinery to drive multi-faceted immune responses at cell and tissue level (1). MSC-based therapies have been used in several clinical trials, where more than 32.000 patients in a range of chronic and acute inflammatory conditions including Diabetes mellitus Type I, Rheumatoid Arthritis, Multiple Sclerosis or Graft vs. Host Disease have been enrolled^1^. In line with this, MSC have shown a robust biosafety profile and partial objective responses, displaying in many cases control of immune and inflammatory responses, symptom alleviation and improved quality of life on treated patients (2). These promising data have paved the way to enhance cell manufacturing processes, reduced burden in clinical scaling and gained comparability between studies that ultimately results in more efficient therapies applied to immune-related disorders. Isolation and expansion of MSC for immune therapy have been successfully achieved from a variety of sources. Among them, MSC derived from human umbilical cord (UC) have raised particular attention due to their high availability and easy access. Noteworthily, given its fetal origin, UC-derived MSC display particular advantages such as improved multipotency, enhanced stemness, and longer proliferation capacity (3). In addition, recent evidence has also suggested stronger immune modulatory effects of UC-MSC *in vitro* and *in vivo* than MSC from classical sources such as bone marrow or adipose tissue (4). Thus, as long as research addressing immune modulatory functions of UC-MSC continues to expand, there will be increasing opportunities to deliver better and more efficient strategies for immune cell therapy.

Current understanding of molecular mechanisms of MSC-driven immune-suppression point to local injury or inflammation as triggers to induce regulatory T cell proliferation/activation, effector T cell anergy, macrophage and dendritic cell modulation or control of metabolic shuffling (5). Proposed mechanisms by which MSC exert immune-suppression are not fully understood, but *in vitro* and *in vivo* data indicate that MSC act on different cell subsets implicated in the onset and maintenance of immune responses at local and systemic level (6). For instance, MSC can restrict proliferation of T and B lymphocytes and suppress their effector activity (7, 8). Moreover, differentiation, antigen presentation and co-stimulation function of dendritic cells as well as inflammatory activity of macrophages are also disrupted in the presence of MSC (9, 10). While there is a debate whether immune suppression mechanisms linked to MSC depend or not on cell contact, there is a broad consensus about the key role that secreted factors play during MSC-mediated immune-suppression. Nevertheless, the large complexity of the cellular and molecular array ruling immune modulation networks by MSC remains unknown, leaving on hold the discovery of new molecular tools with potential application in translational research in the field of MSC-based therapies.

Next generation sequencing (NGS) of whole cell transcriptome has gained exceptional applicability over the past years, in particular when comparative analyses of gene expression in specific experimental settings are required. In the case of MSC, whole transcriptome analyses might have great utility to untangle the complexity of the immune modulatory function by identifying tissue specific cell markers, molecular phenotypes of different MSC subpopulations and assessing the activation of gene networks in pathophysiological settings. Despite the significant role of NGS as powerful tool to understand global gene expression profiles in MSC biology, few reports have addressed MSC identity and function in regard to their tissue origin and functional status (11). Even less studies involving whole transcriptome analyses have explored the molecular mechanisms underlying immune modulation processes by MSC (12). Thus, incremental use of tools such as NSG integrated with a reliable and reproducible immune assay, will significantly contribute to further dissect molecular pathways and discover new links of MSC in the context of immune regulation, all together to improve MSC based therapies.

Here we validated an *in vitro* system to measure diverse facets involved in UC-MSC-triggered immune modulation. Its reliability allowed us to measure and integrate whole secretome and transcriptome in order to corroborate already-known molecular pathways related to MSC-mediated immune modulation. Interestingly we identified novel candidates for the control of inflammation and immune activation by UC-MSC.

## Materials and Methods

### UC-MSC and PBMNC Isolation and Culture

Umbilical cord samples used in this study were obtained after a written consent previously approved by the local ethics committee was signed by UC donors. UC was collected aseptically from women after full-term pregnancy (caesarean section or normal vaginal delivery) as previously described (13). In brief, the UC was cut into 3 cm pieces and residual blood was washed three times with sterile phosphate-buffered saline (PBS) 1X (Gibco, Life Technologies, Carlsbad, CA, United States) containing 1% penicillin/streptomycin 10,000 U/mL (Gibco, Life Technologies, Carlsbad, CA, United States). Right after, each piece of cord was slit longitudinally, cord vessels were removed and the epithelial layer was dismissed. The Wharton’s jelly was removed and cut directly placed in 35 mm tissue culture plastic dishes, containing Dulbecco’s Modified Eagle’s medium (DMEM) with low glucose (Gibco, Life Technologies, Carlsbad, CA, United States) supplemented with 10% human platelet lysate (hPL). These cultures were maintained at 37°C in a humidified atmosphere with 5% CO_2_. Upon reaching 80% confluency, cells were released from the plate using trypsin 0.25% (Gibco, Life Technologies, Carlsbad, CA, United States) for 3 min, counted, and replated. The isolated UC-MSC were characterized by flow cytometry using the surface markers CD90, CD73, CD105, CD45, CD34, HLA-DR (BioLegend, San Diego, United States). Using this protocol we have previously obtained above 95% of cell purity displaying mesenchymal phenotype (13). Stem cell differentiation assays for the three mesenchymal lineages -adipogenic, osteogenic, and chondrogenic- were assessed for each donor. Human peripheral blood mononuclear cells (PBMNC) were isolated from four healthy donors by Ficoll Paque (GE Healthcare) density gradient separation and cryopreserved according to institutional standard protocols. Cells were cryopreserved in 60% medium, 30% fetal bovine serum (FBS) (Gibco, Life Technologies, Carlsbad, CA, United States), 10% DMSO and stored at −190°C.

### Cell-Contact and Indirect UC-MSC/PBMNC Coculture

Two UC-MSC/PBMNC coculture settings were evaluated: direct (cell-to-cell contact) and indirect (transwell) co-cultures. PBMNC were thawed, cultured during 24 h to promote cell stabilization and further used them in immune assays. In order to set up the best conditions for immune stimulation, we performed dose-response curves using known concentrations of PHA and established the best dose for further immune assays (1 ug/mL). PHA concentrations above 3 ug/mL resulted in enhanced T cell mortality due to cell exhaustion. Concentrations below 0,5 ug/mL on the other hand did not show optimal T cell responses. For TCR-dependent T cell activation, anti-CD2, -CD3, -CD28 (αCD3/CD28) T cell activation beads (Miltenyi Biotec GmbH, Bergisch Gladbach, Germany) were used following manufacturer’s instructions. UC-MSC, up to passages 6, were adjusted to 5 × 10^4^ cells/well in a 24-well plate and cultured for 5 h. After this period of time, UC-MSC medium was removed and 5 × 10^5^ PBMNC in RPMI-1640 supplemented with 10% FBS cells were added. Incubation times for all immune assays (ranging from 72 up to 120 h) were also standardized, resulting in a maximum effect at 120 h post MSC co-culture (highest suppression of T lymphocyte proliferation). For indirect co-cultures, UC-MSC were seeded at the bottom of the well and pre-stimulated PBMNC were seeded on top of transwell inserts (1 μm pore size). Each assay was repeated three times and supernatants were collected and stored at −80°C for the subsequent cytokine assays. The inhibitory effect of UC-MSC on lymphocyte proliferation was measured by CD3 positive counts using *CountBright* Absolute Counting Beads (Invitrogen, Life Technologies, Carlsbad, CA, United States). The number of cells per microliter was calculated based on the number of counted cells x concentration of beads/number of counted beads.

### Relative Expression by qRT-PCR

Total RNA of UC-MSC cocultured with PHA or αCD3/CD28-activated PBMNC (in transwell) was isolated by PureLink RNA Mini Kit (Thermo Fisher Scientific, Waltham, MA, United States) according to protocol from manufacturer. RNA concentration and quality were assessed in a Nanodrop-1000 instrument (Nanodrop Technologies, Wilmington, DE, United States). Complementary DNA was prepared by reverse transcription of total RNA with SuperScript^TM^ IV First-Strand cDNA Synthesis Reaction (Invitrogen, San Diego, CA, United States), followed by qRT-PCR in a 7500 Fast Real-Time PCR System (Applied Biosystems, CA, United States) using TaqMan gene expression assays (Applied Biosystems, CA, United States). Analyzed genes included inducible nitric oxide synthase gene (iNOS, Hs01075529_m1) and indoleamine 2,3 dioxygenase (IDO, Hs00984148_m1). In order to normalize mRNA expression, we tested different housekeeping genes that could be effectively used as housekeeping genes in UC-MSC. While HPRT and 18s displayed late CT values and heterogenicity of gene expression, beta-actin did exhibit consistent expression values among MSC from diverse origins (data not shown). Therefore, we employed β-actin (Hs01060665_g1) as housekeeping gene. Briefly, 2 μl of cDNA were used in a PCR reaction volume of 8 μl, containing 5 μl of Gene Expression Master Mix (Applied Biosystems), 0,5 μl of TaqMan probe and 2,5 μl of molecular grade water. All reactions were performed in triplicate, with non-template control for each gene. For each sample, relative gene expression of the target mRNA was calculated, relative to an endogenous reference gene or housekeeping (ΔCq_sample_: Cq_target_- Cq_hkg_). PCR efficiency for each gene was determined based on calibration curve using the formula E = 10^[–1/*slope]*^−1. Relative expression was subsequently calculated using 2^–ΔΔ*CT*^ and Pfaffl methods.

### RNA Sequencing and Transcriptome Analysis

In order to assess transcriptome patterns of UC-MSC in immune modulation, three experimental conditions were stablished: C1, consisting of UC-MSC grown in basal medium; C2, in where UC-MSC were co-cultured with PHA-activated PBMNC (in transwell); and C3 in which UC-MSC were stimulated with PHA. Eight biological replicates were sequenced for each condition for a total of 24 transcriptomes. RNA was extracted using the TRIzol reagent (Life Technologies, Carlsbad, CA, United States) following manufacturer’s instructions and RNA quality was evaluated by the Agilent Bioanalyzer 2100 system. Only preparations with RIN (RNA integrity number) values above 9 were considered. Singled-end 100 bp sequencing was carried out on HiSeq 2500 (Illumina) at Transcriptome and Genome Analysis Laboratory (TAL, Universitaetsmedizin Göttingen, Germany). Raw reads were quality checked with FastQC and then mapped against the Human Reference Genome GRCh38 (Ensembl 92 version) using STAR (v2.6.0) (14, 15). Alignment files were manipulated with Samtools (16), gene expression quantification was conducted with RSEM (17) and differential expression was calculated with EBSeq (18). Briefly EBseq, identified five possible expression patters (based on which conditions genes are equally expressed and which conditions are not), and computed *a posteriori* probability (MAP) to identify the expression pattern that fits better for each gene, and *a posteriori* probability that not all conditions are equally expressed. Differentially expressed genes (DEG) were defined considering a false discovery rate (FDR) with a significance threshold of *p* < 0.05. Functional annotation of DEG, GO enrichment and clustering analyses were performed in R (19) with org. Hs. e.g., Db (20), clusterProfiler (21), and factoextra (22) packages, respectively.

### Luminex Cytokine Assays

Concentrations of cytokines, chemokines and growth factors in cell supernatants were evaluated by cytokine bead array technology. The cytokines (GM-CSF, IL-1β, IL-6, IL-8, TNF-α, IFNγ, IL-2, IL-2R, IL-7, IL-12, IL-15, and IL-17), anti-inflammatory cytokines (IL-1RA, IL-4, IL-5, IL-10, IL-13, and IFN-α), chemokines (eotaxin, IP-10, MCP-1, MIG, MIP-1α, MIP-1β, and RANTES), pro-angiogenic (VEGF), and growth factors (EGF, HGF, bFGF, and G-CSF) were measured by the Human Cytokine 30-plex Assay (Invitrogen, Carlsbad, CA, United States). The procedure was performed according to the manufacturer’s instructions and the concentration was reported in pg/mL.

### CD3^+^ and CD14^+^ T Cell Isolation

Peripheral blood mononuclear cells were thawed and CD3^+^ or CD14^+^ populations were isolated by positive selection using magnetic anti-CD3 and anti-CD14 microbeads (Miltenyi Biotec GmbH, Bergisch Gladbach, Germany), respectively. Following isolation, CD3^+^ or CD14^+^ cells were cultured in RPMI-1640 medium supplemented with 10% FBS and maintained at 37°C and 5% CO2 for 24 h before the assays. Population purity was determined by immune staining with anti-CD14 and anti-CD3 monoclonal antibodies (Miltenyi Biotec GmbH, Bergisch Gladbach, Germany) and flow cytometry analysis.

### Monocyte Conditioning

Isolated CD14^+^ monocytes were pre-conditioned by activated UC-MSC-PBMNC cocultured using transwell system. Briefly, αCD3/CD28-activated CD14 negative fraction in the absence (control) or presence of UC-MSC were seeded at the bottom 24-well plates. Isolated CD14^+^ fractions were added to 1 μm-pore transwell insert. Assays were incubated for 120 h at 37°C with 5% CO2. Following incubation, pre-conditioned CD14^+^ (control and UC-MSC-conditioned) cells were harvested for phenotype characterization and quantification of soluble factors and T cell stimulations. For that, CD14^+^ cells were added to freshly isolated autologous CD3^+^ and exposed to αCD3/CD28T activation for additional 120 h. The effect of preconditioned CD14^+^ fraction on CD3^+^ proliferation was determined by measuring T cell proliferation as described above.

### Flow Cytometry

Umbilical cord-mesenchymal stromal cells pre-conditioned or control CD14^+^ cells were harvested and stained with fluorochrome-labeled monoclonal antibodies against CD206, CD163, and CD301. For T cell subset immune phenotype, cells were stained for CD3, CD4, CD8, CD62L, and CD45RA. CD3^+^CD4^+^ and CD3^+^CD8^+^ cells were gated into naive (TN, CD62L^+^CD45RA^+^), central memory (TCM, CD62L^+^CD45RA–), effector memory (TEM, CD62L–CD45RA–), and terminal differentiated T cells (TEMRA, CD62L–CD45RA^+^). PD-1 expression was assessed in CD3^+^ cells after 120 h in activated-PBMNC-UC-MSC co culture with UC-MSC. For UC-MSC identity and functional phenotyping, cells were cultured and stimulated to different conditions such as PHA, αCD3/CD28 or cytokine cocktail consisting of IL-1b (50 ng/mL) and TNF-a (50 ng/mL) or activated-PBMNC, and evaluated for the expression of PDL-1, PDL-2, CD80, and CD86. For all flow cytometry analyses, isotype control was included. FACSCanto II instrument (Becton Dickinson, San Jose, United States) was used and data set was analyzed using the FlowJo vX.7.0 data analysis software package (TreeStar, United States).

### Statistical Analysis

In order to determine statistic significances, we used *t* student tests and ANOVA for parametric data and Kruskal–Wallace test for non-parametric data. Level of significance was considered when *p*-value was below 0.05. Statistical analysis was carried out using the GraphPad Prism version 6.0 software (La Jolla, CA, United States). Associations between expression patterns of secreted factors in supernatants of PBMNC/MSC cocultures were assessed by Principal Component Analyses (PCA). Hierarchical clustering for heatmap graphical representation was calculated by pairwise distances and clustering method was defined by Pearson correlation using the ClusViz tool (23). Non-linear multi-dimensional visualization based on two-dimensional projection of multivariate data (RadViz) was run in the data mining package Orange 3.0 (Bioinformatics Lab, University of Ljubljana, Slovenia).

## Results

### UC-MSC Abrogate T Cell Proliferation in Cell Contact Dependent and Independent Manner

**FIGURE 1:**
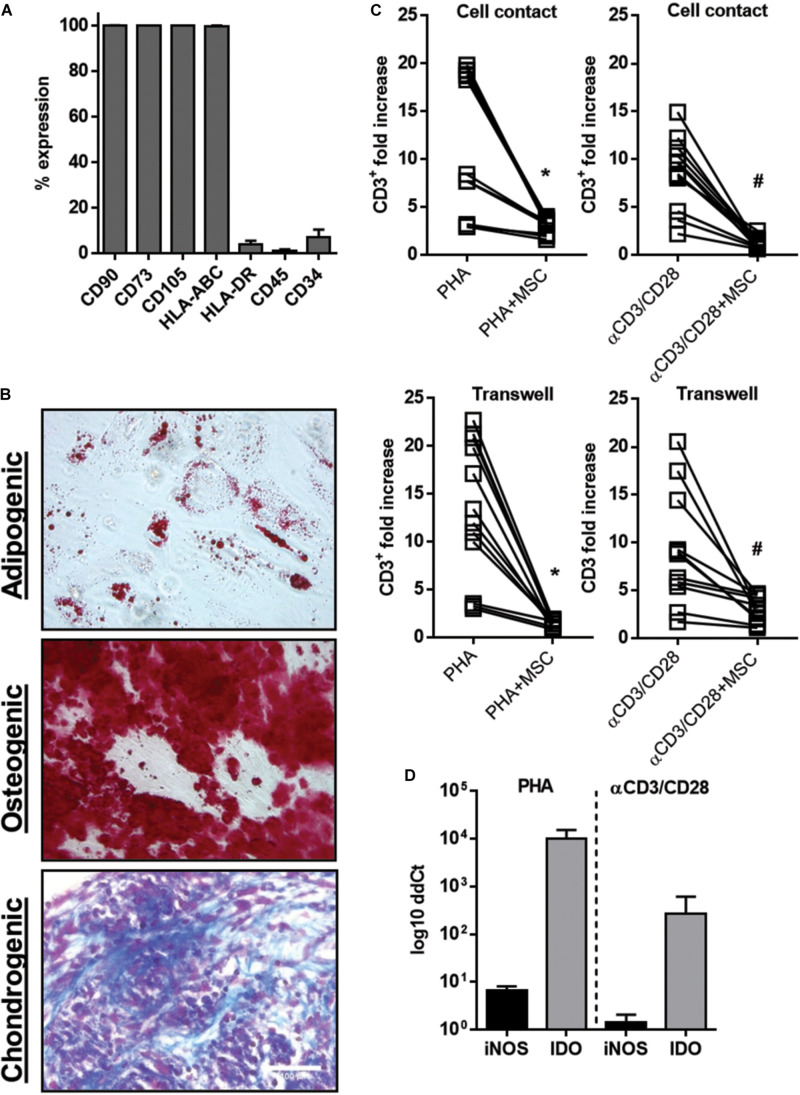
UC-MSC inhibit T cell proliferation induced by PHA and TCR stimulation. **(A)** Percentage of UC-MSC expressing the cell markers CD90, CD73, CD105, HLA-AB, HLA-DR, CD45, and CD34 (*n* = 4). **(B)** Representative micrographies of adipogenic, osteogenic, and chondrogenic differentiation of UC-MSCs in passage 4–5 (*n* = 4 donors). MSCs were induced to differentiate toward osteogenic lineage and verified by alizarin red staining, adipogenic lineage and verified by Oil Red and chondrogenic lineage, verified by alcian blue (magnification = 100×). **(C)** PBMNC (*n* = 4 donors) were stimulated for 5 days with PHA (1 μg/ml) or anti-CD2, -CD3, -CD28 (αCD3/CD28) in presence of UC-MSC (*n* = 3) in a ratio of 10:1 (PBMNC:UC-MSC). Cells were co-cultured in direct contact or in a transwell system, collected and stained with an anti-CD3 antibody. Absolut CD3 counts were determined by counting beads in a flow cytometer. Fold increase ratio of CD3^+^ proliferation was calculated relatively to cell numbers of untreated controls. Data is represented in mean ± SEM. *PHA vs. PHA+UC-MSC, *p* = 0.0001; # αCD3/CD28 vs. αCD3/CD28+UC-MSC, *p* < 0.00001. **(D)** RNA from UC-MSC in transwell experiments was collected. Thereafter, a real-time RT-PCR was performed to quantify indolamine-2,3-dioxygenase (IDO) and inducible NO synthase (iNOS) levels. Log10 ddCt values were calculated relative to UC-MSC in basal culture condition.

To asses the identity of each donor-derived UC-MSC we initially tested the expression of MSC canonical surface markers. Above 95% of the overall isolated cell population in UC donors displayed positive expression of CD90, CD105, CD73, and MHC class I markers and absence of the hematopoietic markers CD34, CD45, and HLA-DR (Figure 1A). We also confirmed their differentiation into adipocytes, chondrocytes, and osteocytes (Figure 1B). In order to assess UC-MSC-driven immune modulatory responses, we first established a coculture immune assay using peripheral blood mononuclear cells (PBMNC). Lymphocyte proliferation/activation was induced either indirectly via phytohemagglutinin (PHA) stimulation or specifically by T cell receptor activation using bead-coated anti-CD2/CD3/CD28 (αCD3/CD28) antibody-coated beads. We tested the suppressive potential of UC-MSC by exposing PHA or αCD3/CD28-stimulated PBMNC to UC-MSC in a ratio 10:1 (PBMNC:UC-MSC) during 5 days in two culture formats: activated PBMNC were seeded directly on UC-MSC monolayer (Cell contact) or alternatively seeded on 1 μm-pore transwell inserts and exposed to UC-MSC monolayers (Transwell). Total CD3^+^ counts were calculated following 5 days in order to measure T cell proliferation. Overall, upon PBMNC stimulation CD3^+^ T cells reached expansion ranging from 2.5 to 24.5-fold increase levels with respect to unstimulated controls in a donor dependent manner (Figure 1C). T cell proliferation responses to PHA or αCD3/CD28 were similar in both transwell inserts and well plates. Importantly, in the presence of UC-MSC we observed dramatic suppression of T cell proliferation in both stimulation setups regardless if T cells were directly in contact with UC-MSC or in contact-independent format. We further verified the activation of key factors reported as mediators of MSC immunosuppressive effects including inducible nitric oxide synthase (iNOS) and indoleamine 2,3-deoxygenase (IDO). UC-MSC IDO and iNOS mRNA levels were measured by real-time PCR under transwell coculture condition and compared with control MSC. Although both IDO and iNOS expression levels were induced in MSC cocultures, PHA stimulation led to significantly higher levels of IDO expression than iNOS in UC-MSC (*p* < 0.05, Figure 1D). Altogether these results confirm that under PHA or αCD3/CD28-induced immune activation, UC-MSC trigger potent and robust immune suppression either in a cell-to-cell interaction or in cell-contact independent setting.

### Analyses of Soluble Factors Present in UC-MSC/PBMNC Cocultures Are Consistent With an Immune Modulatory Secretome

**FIGURE 2:**
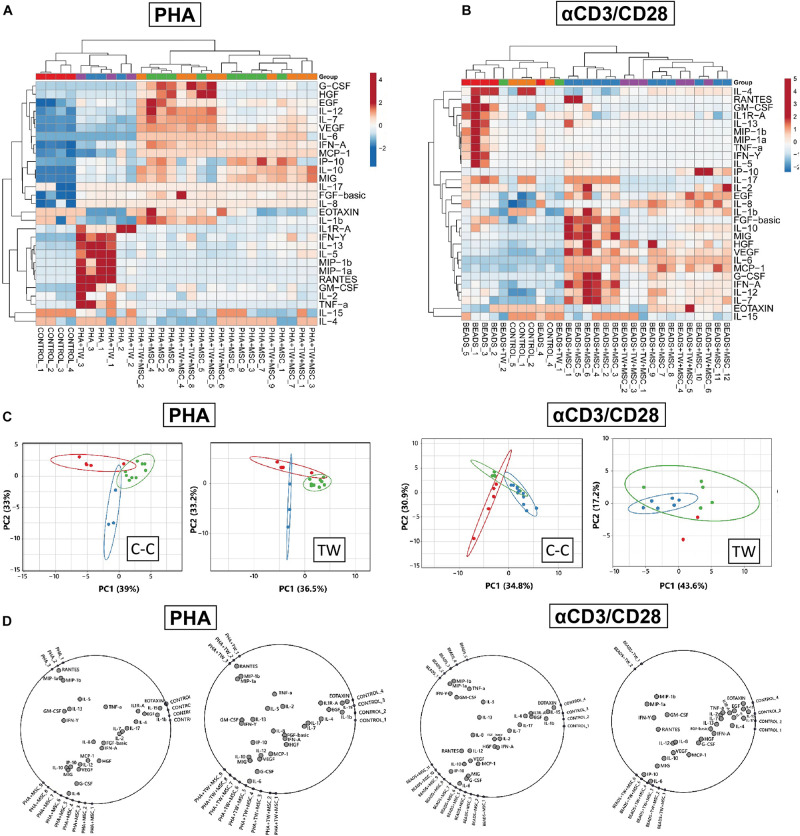
Characterization of immune modulatory secretome in UC-MSC/PBMNC cocultures. Global differences in cytokine concentrations in supernatants from UC-MSC/PBMNC as compared to activated and resting PBMNC in cell-contact and transwell experimental settings were visualized by unsupervised hierarchical clustering in **(A)** PHA-stimulated and **(B)** αCD3/CD28-stimulated UC-MSC/PBMNC cocultures. **(C)** Two-dimensional Principal Component Analyses (PCA) was carried out to determine associations between expression patterns of analyzed factors in PHA and αCD3/CD28 cocultures. **(D)** Radial visualization (RadViz) analyses showing factors assigned to dimensional anchors placed on the circumference of each circle for PHA and αCD3/CD28 stimulations in cell contact and transwell formats.

In order to detect molecules associated to UC-MSC-mediated immune suppression of T cells, we further conducted analyses of soluble factors present in activated UC-MSC/PBMNC cocultures. We focused on the specific expression of Th1/Th2 cytokines, chemokines and growth factors tightly associated to innate and adoptive immune responses upon PHA and αCD3/CD28 stimulation. Global differences in cytokine accumulation from activated PBMNC/UC-MSC cocultures, compared to activated and resting PBMNC were visualized by unsupervised hierarchical clustering and Pearson’s Correlation. We found that supernatants from activated PBMNC in presence of UC-MSC clustered apart from stimulated and rested PBMNC in both PHA and αCD3/CD28 cultures, confirming a unique pattern of cytokine/chemokine/growth factor secretion signature during MSC-mediated immune suppression (Figures 2A,B). Interestingly, there were not striking differences in clustering patterns between cell-contact and transwell formats, suggesting similar secretion responses mediated by UC-MSC regardless of cell contact-dependent processes. The association between expression patterns of analyzed factors was further assessed by Principal Component Analyses (PCA). In two-dimensional analyses, first and second principal components explained 33 and 39% associations for PHA, 33.2 and 36.5% for PHA-TW, 30.9 and 34.8% for Beads and 17.2 and 43.6% for Beads-TW. Three different clusters, “control” (green), “stimulated” (red), or “stimulated+MSC” (blue) were observed in all stimulation formats except for αCD3/CD28-activated group in TW format, where no clear distinction between these conditions was detected (Figure 2C). To individualize those factors prompting to cluster separation in PHA or αCD3/CD28 stimulation under cell-contact dependent and independent settings, we applied a radial visualization tool (RadViz). Here, all observations in each experimental group were assigned to dimensional anchors (DA) and placed on the circumference of a circle. Location vectors within the circle for each cytokine/chemokine/growth factor were calculated as a function of their relative association to each experimental DA, revealing factor arrangements that influence cluster separation for each condition. In the case of PHA-stimulation we observed that MIP-1a, MIP-1b, GM-CSF, RANTES, TNFα, or IFNγ predominantly associated to immune-activated PBMNC (Figure 2D), whereas IL-6, G-CSF, MIG, IL-10, or IP-10 seemed to be clustered in the presence of UC-MSC in both cell-contact and transwell formats. Similar results were observed after αCD3/CD28 stimulation in cell-contact format, where comparable patterns of factor association upon stimulation were evidenced. Although, αCD3/CD28 stimulation in the transwell format did not show a strong pattern of cytokine/chemokine association to PBMNC stimulation or MSC/PBMNC cocultures, we observed a pattern reflecting a similar secretion response as found after PHA-stimulation. Taking together, multiparametric analyses of secretome comparing immune-activated PBMNC and PBMNC/UC-MSC cocultures allowed us to identify potential patterns of cytokine/chemokine expression that are strongly linked to immune modulatory pathways triggered by activated UC-MSC. Overall, these results suggest that under multifaceted inflammatory challenge, UC-MSC can dramatically alter the pattern of accumulation of several soluble factors, possibly leading to the suppression of T cell proliferation.

### Transcriptome Signature of UC-MSC Under Experimental Inflammation Reveals New Mechanisms of MSC-Driven Immune Modulation

**FIGURE 3:**
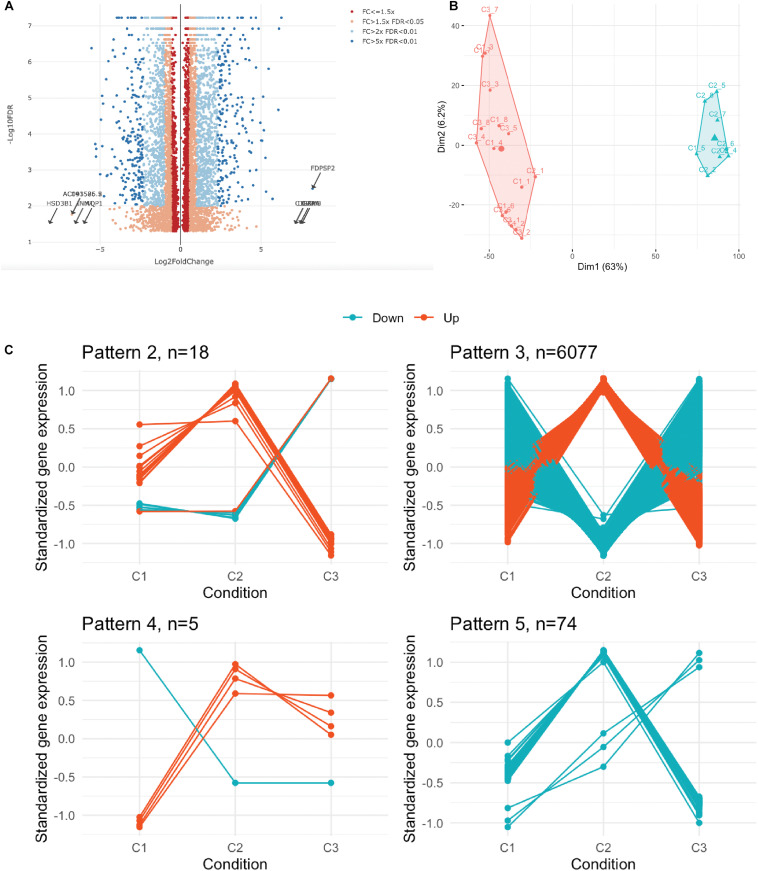
Transcriptome profiling of UC-MSC under experimental inflammation. **(A)** Volcano plot showing the distribution of all differentially expressed genes. FC, fold change; FDR, false discovery rate. **(B)** Hierarchical clustering of the 24 transcriptomes based on the normalized counts of differentially expressed genes. **(C)** Standardized gene expression of DEG in each condition grouped by its identified expression pattern.

**FIGURE 4:**
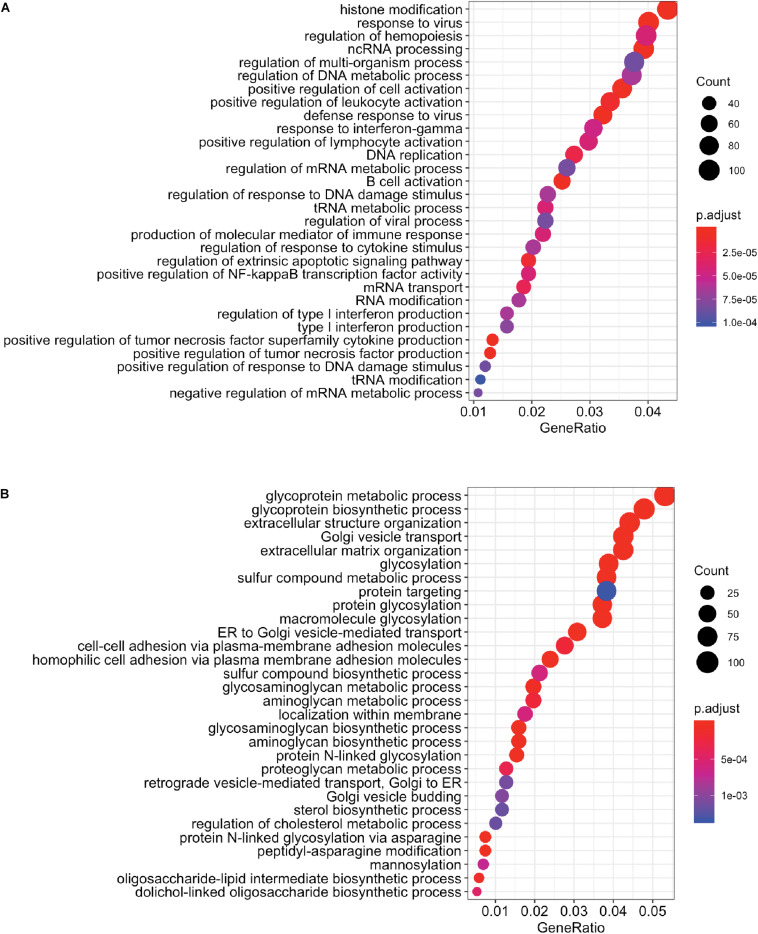
GO term enrichment analysis by biological process. Enrichment analysis for **(A)** upregulated (*n* = 3,227) and **(B)** downregulated (*n* = 2,850) DEG in pattern 3 are presented (*p* < 0.05). Terms are presented from highest to lowest combined score (a combination of the *p*-value and *z*-score) calculated as part of Enrichr analysis.

We next performed whole transcriptome analysis of UC-MSC under experimental inflammation in order to unveil new molecular pathways driving immune modulation by UC-MSC. We chose PHA stimulation on transwell system based on previous secretome analyses, showing a clear clustering pattern between groups. Whole cell transcriptomes from unstimulated UC-MSC (*n* = 8, C1), PHA-stimulated UC-MSC/PBMNC cocultures (*n* = 8, C2), and PHA-stimulated UC-MSC controls (*n* = 8, C3) were obtained. In total, 6,174 genes showed differential expression (Figure 3A), being 3,227 upregulated and 2,850 downregulated genes (FDR-adjusted, *p* < 0.05) when C2 and C1 were compared (Supplementary File 1). About 42% of these DEG showed a fold change greater than 2 (C2/C1), indicating that PHA activation of PBMNC led to significant changes in UC-MSC transcriptome. Hierarchical clustering analysis based on the normalized counts of DEG, grouped sequenced transcriptomes in two clear groups, one composed by C2 and other where C1 and C3 were grouped together (Figure 3B and Supplementary Figure 1). During differential expression analysis, five possible expression patterns were enumerated: in pattern 1, there were no DEG in C1, C2 and C3; pattern 2 grouped those DEG present only in C3 but not in C1 and C2; pattern 3 indicated DEG present in C2 but not in C1 or C3; pattern 4 corresponded to DEG in C2 and C3 but not in C1; and pattern 5 in were DEG were present in C1, C2, and C3. Remarkably, most of DEG (98.4%) fell into expression pattern 3 (Figure 3C) in where no significant differences between C1 and C3 were observed, while significant expression changes were detected in C2. In order to narrow down the selection of potential DEG candidates involved in MSC immune modulation, we chose genes that displayed more than 1.5 fold-change in PHA-stimulated cocultures related to untreated MSC (C3/C1 > 1.5) and 1.0 fold-change in PHA-treated PBMNC/UC-MSC cocultures related to untreated MSC (C2/C1 = 1) (Supplementary File 1). We found 131 upregulated genes and 269 downregulated genes in PHA-stimulated UC-MSC/PBMNC cocultures. Among them, we further looked over the expression profiles of 47 DEG for C1, C2, and C3, including chemokines, anti-inflammatory and pro-inflammatory cytokines, growth factors, adhesion molecules, and other genes previously reported as key molecules in the control of immune responses by MSC. We found that C2 showed upregulation of 36 (76.6%) genes as compared to C1, while 11 (23.4%) were down-regulated (Table 1). Finally, GO term enrichment analysis of up and downregulated DEG found in pattern 3 was conducted in order to identify gene clusters involved in biological processes (Figures 4A,B) cellular components and molecular functions (Supplementary Figures 2, 3 and Supplementary File 2). GO enrichment analyses evidenced the activation of several molecular pathways related to protein synthesis and secretion at endosomal/exosomal cell compartments, implying intense secretory processes going on in UC-MSC under immune modulation. Importantly, most of the enriched terms in overexpressed genes related to biological processes were linked to major immune regulation pathways (e.g., response to virus, positive regulation of leukocyte activation, B cell activation, positive regulation of tumor necrosis factor production), suggesting an intimate relation of immune responses and gene expression in UC-MSC that sheds light into this immunological function and opens an opportunity to explore new genes involved in UC-MSC-mediated immune modulation.

**TABLE 1:** Selected DEG involved in immune modulation by MSC.

Ensembl gene ID	Gene	Category	C1 (TPM*)	C2 (TPM)	C3 (TPM)	C2/C1
ENSG00000134460	IL-2R	Pro-inflammatory cytokines	0,24	41,20	0,38	170,89
ENSG00000100368	CD131	Immunomodulation	0,44	5,49	0,45	12,42
ENSG00000277632	MIP-1a	Chemokines	2,43	18,89	0,70	7,77
ENSG00000169245	IP10	Chemokines	231,86	1.796,86	1,00	7,75
ENSG00000131203	IDO1	Immunomodulation	1.019,58	6.739,63	4,06	6,61
ENSG00000136634	IL-10	Anti-inflammatory cytokines	0,10	0,63	0,00	6,46
ENSG00000135077	Tim-3	Immunomodulation	4,69	24,00	4,73	5,11
ENSG00000162692	CD106	Adhesion molecules	470,45	2.262,98	323,86	4,81
ENSG00000138755	MIG	Chemokines	2.975,08	13.407,50	8,90	4,51
ENSG00000120217	CD274	Immunomodulation	2.218,85	8.963,76	721,68	4,04
ENSG00000164342	TLR-3	Immunomodulation	90,33	358,27	44,51	3,97
ENSG00000164400	GM-CSF	Pro-inflammatory cytokines	92,75	327,40	72,28	3,53
ENSG00000090339	CD54	Adhesion molecules	19.571,90	67.853,90	6.938,78	3,47
ENSG00000136869	TLR-4	Immunomodulation	4,34	14,56	2,78	3,36
ENSG00000168811	IL-12A	Pro-inflammatory cytokines	22,85	67,73	16,43	2,96
ENSG00000172156	Eotaxin	Chemokines	0,21	0,61	0,40	2,94
ENSG00000064300	CD271	Immunomodulation	6,89	18,19	3,80	2,64
ENSG00000007171	INOS	Immunomodulation	11,40	28,95	0,59	2,54
ENSG00000232810	TNF	Pro-inflammatory cytokines	0,75	1,88	0,27	2,51
ENSG00000108342	G-CSF	Growth factors	1.282,41	3.092,31	892,93	2,41
ENSG00000164136	IL-15	Pro-inflammatory cytokines	182,61	436,09	157,91	2,39
ENSG00000108691	MCP1	Chemokines	2.766,09	6.329,78	2.195,54	2,29
ENSG00000136689	IL-1Ra	Anti-inflammatory cytokines	3,26	6,53	3,34	2,00
ENSG00000169429	IL-8	Pro-inflammatory cytokines	31.530,40	62.329,00	32.269,30	1,98
ENSG00000138685	FGF-basic	Growth factors	29.847,00	58.499,40	27.953,70	1,96
ENSG00000170017	ALCAM	Adhesion molecules	2.834,57	5.541,63	2.820,10	1,96
ENSG00000271503	RANTES	Chemokines	634,14	1.234,73	36,63	1,95
ENSG00000112715	VEGF	Growth factors	11.938,60	21.878,40	9.581,04	1,83
ENSG00000026508	CD44	Adhesion molecules	9.061,18	16.467,30	9.987,75	1,82
ENSG00000163739	CXCL1	Immunomodulation	7.007,11	12.338,30	7.225,25	1,76
ENSG00000136244	IL-6	Pro-inflammatory cytokines	11.512,00	18.228,30	12.600,00	1,58
ENSG00000125538	IL-1b	Pro-inflammatory cytokines	18.222,10	26.799,80	20.471,00	1,47
ENSG00000113520	IL-4	Anti-inflammatory cytokines	0,07	0,10	0,00	1,45
ENSG00000103855	CD276	Immunomodulation	2.553,27	1.670,55	2.904,12	−1,53
ENSG00000197919	IFN-a	Anti-inflammatory cytokines	46,50	22,77	73,31	−2,04
ENSG00000169439	CD362	Immunomodulation	2.059,37	976,69	2.397,84	−2,11
ENSG00000113302	IL-12B	Pro-inflammatory cytokines	0,28	0,12	0,52	−2,32
ENSG00000076706	CD146	Immunomodulation	1.400,11	531,04	1.400,00	−2,64
ENSG00000138798	EGF	Growth factors	325,45	100,18	465,06	−3,25
ENSG00000019991	HGF	Growth factors	1.219,34	272,46	1.266,22	−4,48
ENSG00000017427	IGF1	Immunomodulation	28,20	1,59	32,35	−17,75

*TPM, transcripts per million.

### Monocyte Regulation Explains in Part the Potent Immune-Modulatory Effect of UC-MSC

**FIGURE 5:**
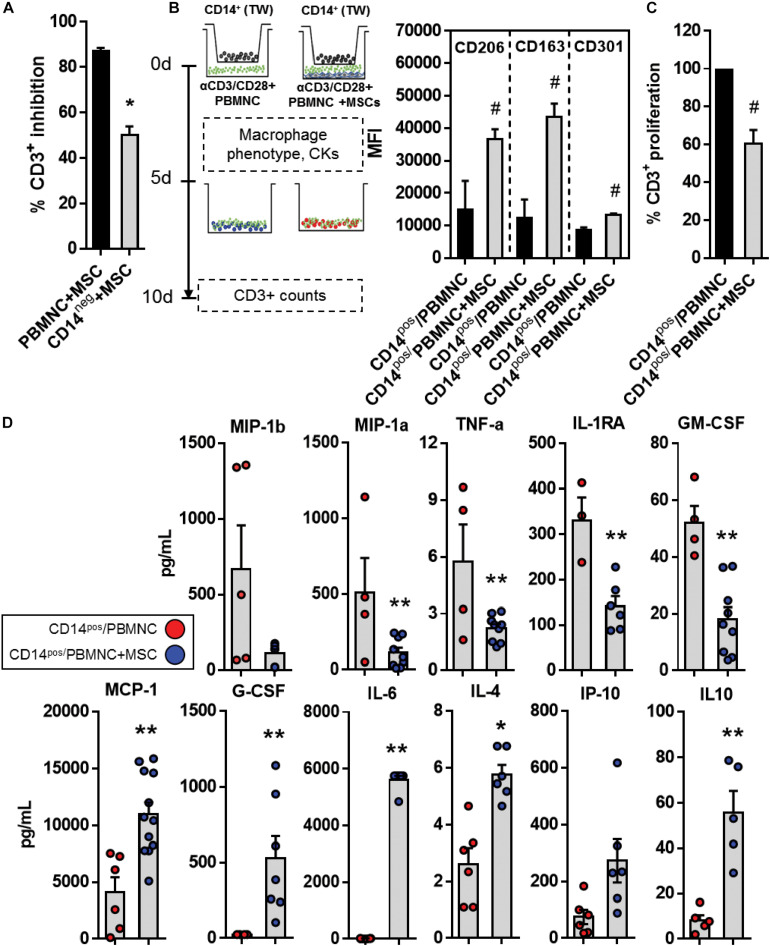
UC-MSC trigger reprogramming of M1 toward M2-Macrophage. **(A)** Percentage of CD3^+^ T cell suppression in αCD3/CD28-activated PBMNC/UC-MSC or monocyte-depleted (CD14^neg^)-PBMNC/UC-MSC cocultures (*p < 0.01, *n* = 3). **(B)** CD14^pos^ monocytes were preconditioned with UC-MSC under experimental inflammation. CD14^pos^ cells were purified, seeded in the upper chamber of 1 μm-pore transwell inserts and exposed to activated autologous PBMNC (CD14^pos^/PBMNC), or activated PBMNC/UC-MSC cocultures (CD14^pos^/PBMNC+MSC) for 5 days. Preconditioned monocytes (CD14^pos^/PBMNC or CD14^pos^/PBMNC+MSC) were further transferred to activated autologous CD3^+^ cells and T cell suppression was determined 5 days post culture. Expression of M2-like markers CD206, CD163, and CD301 in preconditioned CD14^pos^ monocytes at day 5 was determined (# vs. CD14^pos^/PBMNC, *p* = *p* < 0.05, *n* = 3–4). **(C)** Percentage of CD3^+^ T cell proliferation after culture with CD14^pos^/PBMNC or CD14^pos^/PBMNC+MSC monocytes in a ratio 10:1 (CD3^+^:CD14^pos^) (# vs. CD14^pos^/PBMNC, *p* < 0.05, *n* = 3). **(D)** Supernatants from CD14^pos^/PBMNC or CD14^pos^/PBMNC+MSC were collected and analyzed for cytokine, chemokines and growth factor production following preconditioning experiments (** vs. CD14^pos^/PBMNC, *p* = 0 < 0.05, *n* = 4–8).

So far secretome and transcriptome data suggested a pivotal role of inflammation-related cytokines and chemokines that play a direct function on differentiation and activation of myeloid cells in UC-MSC-mediated immune modulation. For instance, a quick look at GO terms enrichment analysis showed upregulated DEG relative to myeloid cell differentiation, macrophage differentiation, and regulation of macrophage differentiation. Concurrently, analyses of secretome under immunomodulation showed accumulation of soluble factors such as G-CSF, MCP-1, IL-10, IL6, IP10 that strongly associate to myeloid function. We therefore assessed differentiation and activation status of monocyte populations in the context of UC-MSC-driven immune modulation. We first depleted CD14^+^ monocytes from PBMNC (CD14^neg^) and performed UC-MSC cocultures under T-cell activation. Following a five-day culture, we observed a significant reduction of T cell suppression in CD14^neg^ cells (51 vs. 87%, CD14^neg^+MSC vs. PBMNC+MSC, *p* = 0.0001, Figure 5A). Since CD14^+^ monocytes might be playing an extra role in UC-MSC-driven T cell suppression, we next investigated whether exposure to immune-modulatory environment driven by UC-MSC would alter CD14^+^ differentiation and function. Isolated CD14^+^ (CD14^pos^) were seeded in 1 μm-pore transwells, exposed to αCD3/CD28-activated PBMNC (CD14^pos^/PBMNC) or PBMNC/UC-MSC cocultures (CD14^pos^/PBMNC+MSC) to evaluate the expression of CD206 (*p* = 0.035), CD163 (*p* = 0.012), and CD301 (*p* = 0.008) markers after 5 days (Figure 5B). PBMNC/UC-MSC conditioned CD14^pos^ cells displayed significant levels of CD206 and CD163 markers, suggesting macrophage type-II (M2) enrichment, as compared to CD14^pos^ exposed to activated PBMNC. We next transferred CD14^pos^ cells conditioned with activated PBMNC (CD14^pos^/PBMNC) or PBMNC/UC-MSC cocultures (CD14^pos^/PBMNC+MSC) to αCD3/CD28-activated CD3^+^ lymphocytes and cultured them for additional 5 days. We observed a significant reduction of T cell proliferation (60.3%, *p* = 0.001) as compared to CD3^+^ cells cultured with PBMNC-conditioned CD14^+^ cells (Figure 5C). Furthermore, we measured levels of cytokines present in supernatants of CD14^pos^ monocytes following preconditioning with activated PBMNC or PBMNC/UC-MSC cocultures. Secretion of M1-type inflammatory cytokines/chemokines from activated PBMNC-conditioned CD14^pos^ cells such as MIP-1a (*p* = 0.061), MIP-1b (*p* = 0.031), TNFα (*p* = 0.018), IL-1RA (*p* = 0.004), and GM-CSF (*p* < 0.000), were significantly induced as compared to CD14^pos^/PBMNC+MSC (Figure 5D). Conversely, CD14^pos^ monocytes exposed to PBMNC/UC-MSC cocultures, displayed enhanced amounts of M2-like cytokines such as IL-4 (*p* < 0.000), MCP-1 (*p* = 0.001), IL-6 (*p* < 0.000), IP-10 (*p* = 0.034), IL-10 (*p* = 0.001), and G-CSF (*p* = 0.033), as compared to CD14^pos^ cells exposed to activated PBMNC. Thus, under inflammatory stimuli, UC-MSC can promote the enrichment of M2-type regulatory monocytes, induce type-II regulatory signals and reduce the availability of M1-type inflammatory cytokines present in the cytokine/chemokine milieu, finally leading to T cell suppression.

### UC-MSC Control the Differentiation and Activation of T Lymphocytes During Immune Modulation

**FIGURE 6:**
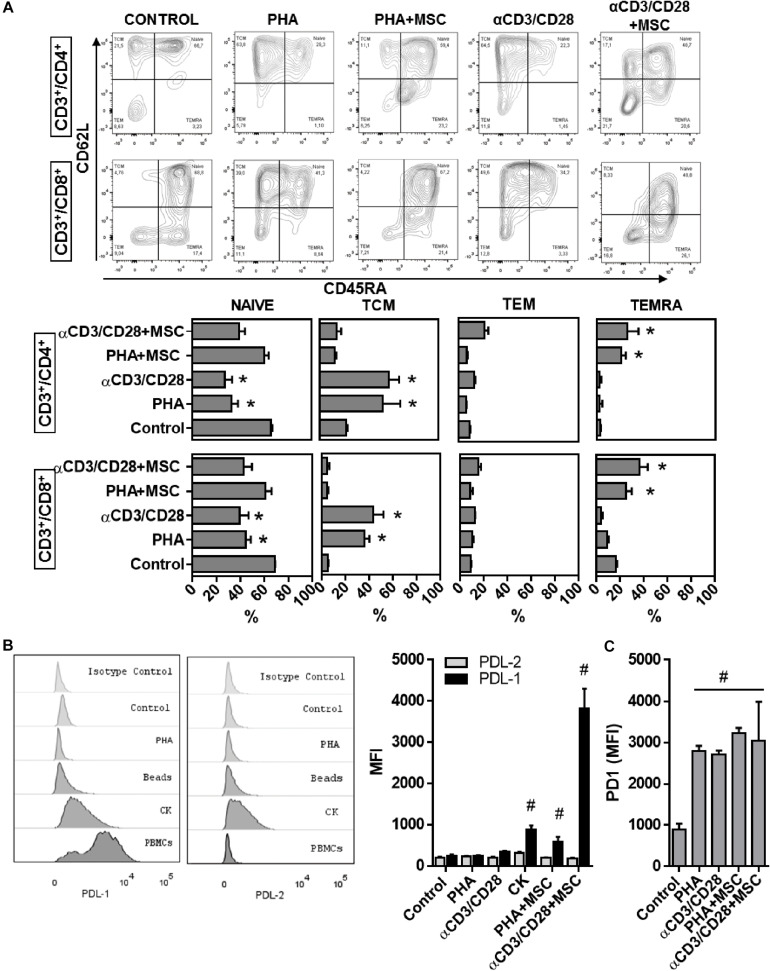
Immune modulatory UC-MSC activate T cell regulatory pathways leading to reduction of activated T cells. **(A)** Flow cytometry analyses and percentages of naïve (NAÏVE, CD45RA^+^/CD62L^+^), central memory (TCM, CD45RA^–^/CD62L^+^), effector memory (TEM, CD45RA^–^/CD62L^–^), and terminal effector (TEMRA, CD45RA+/CD62L^–^) T lymphocyte subsets in CD3^+^/CD4^+^ and CD3^+^/CD8^+^ T cell populations in PBMNC or PBMNC/UC-MSC cocultures activated with PHA or αCD3/CD28 for 5 days (* vs. Control, *p* < 0.05, *n* = 3–5). **(B)** Expression of Programmed cell death protein ligand 1 (PD-L1) and 2 (PD-L2) in UC-MSC, assessed by flow cytometry following 5 days of treatment with PHA, αCD3/CD28, TNFα/IL1b (CK), PHA and αCD3/CD28-activated PBMNC/MSC (# vs. Control, *p* < 0.05, *n* = 6–8). **(C)** Programmed cell death protein 1 (PD-1) expression in CD3+ T cells 5 days stimulation with PHA, αCD3/CD28, TNFα/IL1b (CK), PHA and αCD3/CD28-activated PBMNC/MSC (# vs. Control, *p* < 0.05, *n* = 3–5).

Since UC-MSC-driven monocyte reprogramming accounts for less than half of the capacity of UC-MSC to supress T cells, and whole transcriptome analyses revealed enhanced expression of genes related to immune modulation pathways directly affecting lymphocyte activation/proliferation (Table 1), we postulated a potential direct role of UC-MSC in T cell control under experimental inflammation. Since previous reports have described induction of regulatory T (Treg) cell compartment as hallmark of MSC-driven immunomodulation, we first tested whether UC-MSC were able to induce Tregs. We did not find enrichment of CD3^+^/CD4^+^CD127^–^CD25^high^ cell population in activated PBMNC/UC-MSC cocultures as compared to activated PBMNC (data not shown). We therefore focused in the activation level of T cells by analyzing the differentiation of T cell subpopulations in activated PBMNC/UC-MSC cocultures. Upon PBMNC activation CD4^+^ cells displayed reduction of naïve (CD45RA^+^CD62L^+^) populations from 65.5 to 26.8% after αCD3/CD28 activation (*p* < 0.001) and 33% after PHA treatment (*p* > 0.001, Figure 6A). Conversely, T cell activation led to enhanced frequencies of central memory (TCM, CD45RA^–^CD62L^+^) subpopulations after αCD3/CD28 (from 22.2 to 58.2, *p* < 0.001) or PHA (up to 53.4%, *p* < 0.001), as compared to non-activated controls. However, in the presence of UC-MSC, TCM subsets frequencies in both CD4^+^ cells did not increase upon T cell activation. Remarkably, percentages of terminally differentiated effector memory (TEMRA, CD45RA^+^CD62L^–^) subsets in CD4^+^ cells increased from 2.9 up to 20.7% in the presence of UC-MSC after PHA treatment (*p* < 0.000) and 25.6% after αCD3/CD28 activation (*p* < 0.000). In the case of CD8^+^ cells, naïve populations significantly reduced upon activation with αCD3/CD28 (from 68.9 to 39.3%, *p* < 0.001) or PHA (44.3%, *p* < 0.001) as compared to control. Conversely, CD8^+^ activation led to enhanced frequencies of TCM subsets after αCD3/CD28 treatment or PHA (up to 43.7 and 35.9%, respectively, *p* < 0.001), compared to non-activated controls (5.2%). UC-MSC also enhanced TEMRA subpopulations in CD8^+^ cells during αCD3/CD28-treatment (up to 36.6%, *p* < 0.000) or PHA (up to 25.4%, *p* < 0.000).

Since UC-MSC seem to be triggering regulatory processes on T cell differentiation and activation, we next measured the expression of receptors involved in the immune checkpoint pathway Programmed Death (PD). We first assessed expression of the ligands PD-L1 and PD-L2 in UC-MSC after coculture with PHA and αCD3/CD28-activated PBMNC and observed a significant induction of PD-L1 but not PD-L2 (*p* = 0.005 for PHA- and *p* < 0.000 for αCD3/CD28-activated PBMNC/UC-MSC cocultures, Figure 6B). Changes in the expression of additional molecules such as CD80 and CD86 in UC-MSC-driven immune modulation were not detected (data not shown). Interestingly, incubation with IL-1β and TNFα during 48 h in the absence of activated PBMNC, significantly enhanced the expression of PD-L1 in UC-MSC, as compared to basal control (*p* < 0.0001). We also evaluated expression levels of the PD-L1 receptor PD-1 in PHA and αCD3/CD28-activated T cells under UC-MSC immune modulation. Following activation, T cells maintained enhanced expression of PD-1 120 h post stimulation (*p* < 0.05, for PHA and αCD3/CD28 vs. control, Figure 6C). Enhanced PD-1 expression in T cells was maintained in the presence of UC-MSC. Together these results point to complementary mechanisms involving immunological checkpoint mechanisms displayed by UC-MSC to induce T cell suppression in both CD4 and CD8 lymphocytes.

### Identification and Functional Analyses of Novel Immune Mediators Involved in UC-MSC Modulation

**FIGURE 7:**
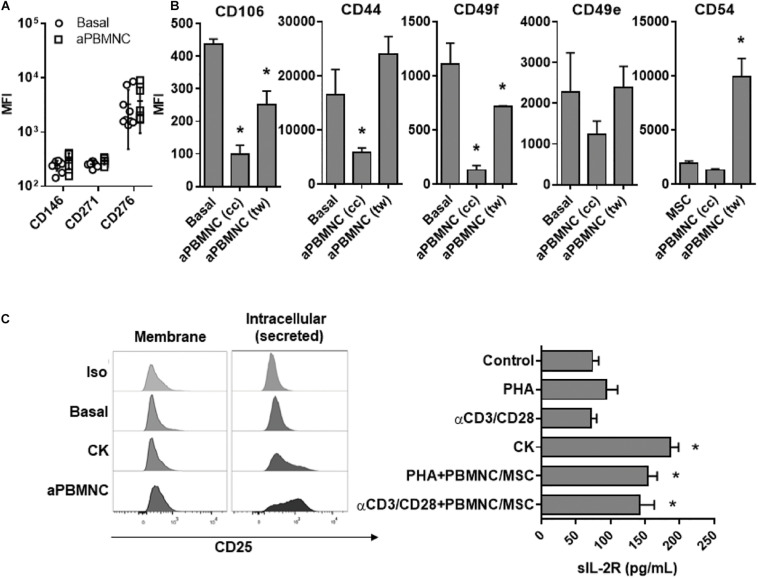
Identification of novel functional markers of immune-modulatory UC.MSC. **(A)** Expression of CD146, CD271, and CD276 in UC-MSC under basal conditions or exposed to activated PBMNC for 5 days. **(B)** Expression of adhesion molecules CD106, CD54, CD44, CD49f, and CD49e in UC-MSC under basal conditions or exposed to activated PBMNC in cell contact (cc) or transwell (tw) setting for 5 days (* vs. Basal, *p* < 0.05, *n* = 4–6). **(C)** Detection of IL-2R in cell membrane, intracellular and in supernatants of UC-MSC treated with PHA, αCD3/CD28, TNFα/IL1b (CK), PHA and αCD3/CD28-activated PBMNC/MSC (* vs. Basal, *p* < 0.05, *n* = 4–6).

Considering the strong potential of UC-MSC for clinical translation, identification of functionally relevant cell surface markers might enable the prediction of immunomodulatory responses of cell-based therapeutics and enhance clinical efficacy for therapy applications. Here, secretome and transcriptome analyses have played a pivotal role in identifying new potential markers with significant relevance for the assessment of immunomodulatory function. We first evaluated the expression of markers to characterize specific MSC subpopulations previously identified (24–26) to harbor immune regulatory effects such as CD276, CD271, and CD146 on UC-MSC subjected to αCD3/CD28-activated PBMNC (Figure 7A). However, we did not find differences on the expression of these markers in immune-activated UC-MSC as compared to basal controls. In a similar experimental setting, we explored the expression of UC-MSC adhesion molecules in basal conditions and compared them to MSC-mediated immune modulation. We found that UC-MSC expressed high levels of CD106, CD44, and CD49 (e and f) but low levels of CD54 under basal conditions (Figure 7B). Interestingly, under PBMNC activation in the cell-contact setting, UC-MSC strongly downregulated CD106 (*p* < 0.000), CD44 (*p* < 0.000), and CD49f (*p* < 0.000). However, only expression of CD106 and CD49f was significantly reduced in UC-MSC (*p* = 0.000) under activated PBMNC coculture in a cell independent format, although reduction of marker expression was less pronounced than the levels found in the cell-contact setting. Interestingly, CD54 was the only adhesion molecule significantly induced in UC-MSC after coculture with activated PBMNC in a cell independent manner (*p* < 0.000).

Finally, taking in account the strong induction of IL-2R gene expression detected in transcriptome analyses (Table 1), we investigated IL-2R expression in UC-MSC following cocultures with immune activated PBMNC or TNFα/IL-1β stimulation. Surprisingly we did not find expression of IL-2R on cell surface but rather detected high intracellular levels in UC-MSC (Figure 7C). In line with that, supernatants of TNFα/IL-1β, PHA or αCD3/CD28-treated UC-MSC as well as from activated PBMNC/UC-MSC cocultures (PHA or αCD3/CD28) were assessed for the presence of soluble IL-2R. We found significantly higher levels of soluble IL-2R in UC-MSC supernatants exposed to inflammatory cytokines (186.1 pg/mL, *p* < 0.000), PHA-activated PBMNC cocultures (153.5 pg/mL, *p* = 0.001), and αCD3/CD28-activated PBMNC cocultures (141.5 pg/mL, *p* = 0.037) as compared to base line (73.7 pg/mL). These results suggest a potential key role of secreted IL-2R in immune modulation by UC-MSC. Altogether, these data point to adhesion molecules and cytokine receptors not only as novel molecular pathways of great interest to understand the immune modulatory mechanisms behind UC-MSC, but also as promising biomarkers to monitor UC-MSC potency in the context of cell therapy.

## Discussion

This report introduces an analytic approach based on the application of combined transcriptome and secretome tools in the context of experimental immune activation, to validate molecular pathways linked to MSC-driven immune modulation and outline new cellular and molecular mechanisms present in human UC-MSC. By taking advantage of this strategy, we evidenced the activation of major regulatory processes triggered by UC-MSC under induced inflammation and T cell activation, that effectively impacted on the remodeling of monocyte and T lymphocyte function, ultimately leading to the control of enhanced immune responses. So far, genome-wide sequencing approaches have not been extensively used to analyze gene expression patterns related to MSC immune biology. Most of reported transcriptome analyses have been restricted to comparative studies addressing MSC identity from diverse origins (27–29). For instance global gene expression of adipose-derived MSC undergoing differentiation, allowed the identification of specific gene expression signatures associated to adipogenic, osteogenic and chondrogenic lineages (30). Transcriptome analyses of TLR4-primed bone marrow MSC have been previously addressed (31), identifying enhanced expression of chemotaxis and inflammation-related genes such as CXCL2, CCL5, CXCL1, CXCL3, CXCL10, IFIT1, IFIT2, IFIT5, ISG15, TNF and prostaglandin family. Gene expression patterns were further associated with enhanced MSC migration via activation of NF-κB and PI3K related to IL-1β, IL-6 and CXCL8 pathways. In a vascularization model using BM-MSC/endothelial cell cocultures (32), RNA sequencing analysis revealed a large proportion of angiogenesis-related factors (26/78 upregulated genes, >2-fold; 5/78 > downregulated genes, >2-fold) differentially expressed and closely linked to NF-kB signaling via IL-1β and IL-6 induction, pointing IL-6 as pivotal immunosuppressive safeguard during MCS-endothelial cell-driven angiogenesis via NF-kB. Finally, in a rat model of corneal transplant, congenic BM-MSC protected corneal allograft from immune rejection, identifying immunological signatures of differentially expressed genes induced by MSC, including CTLA4 and CXCL9 (33). In this work, we introduced a simultaneous approach to evaluate whole transcriptome and secretome analyses of UC-MSC during immune modulation in cells exposed to PHA-activated PBMNC. Using this experimental setting we were able to detect over 6.000 differentially expressed genes present in UC-MSC associated to a variety of biological processes such as regulation of leukocyte activation, cytokine production, membrane adhesion molecules, etc, strongly implicated in the regulation of inflammatory responses. Among these factors, G-CSF, MCP-1, ICAM (CD54), VCAM (CD106), and IL-2Rα (CD25) showed to be key mediators of UC-MSC-triggered immune modulation. In fact, we were able to validate the enhanced expression of these molecules at protein level in further functional experiments. In particular, IL-2Rα was one of the highest upregulated genes found in PHA-treated PBMNC/UC-MSC cocultures. We found not only enhanced intracellular levels of this receptor but also significant accumulation in supernatants from TNFα/IL-1β-treated UC-MSC. Provided that soluble form of IL-2Rα has been described as decoy receptor to supress secreted IL-2 activity *in vitro* and *in vivo* (34), our data suggest an attractive new mechanism for immune suppression by UC-MSC. All these mechanisms appear to tailor localized immunosuppression, consistent with the proposed role of UC-MSC as mediators of immune tolerance.

There is a general agreement about the potent effect of MSC in inducing immune modulation on several immune cell types. Experimental models involving cell culture of activated-PBMNC cells in the presence of MSC from diverse origins have allowed the identification of mechanisms leading to T cell inhibition by MSC. Initial models pointed to enzyme-mediated tryptophan depletion as major inhibitory mechanism of T-cell proliferation, involving mainly endoplasmic reticulum stress and preventing T cell activation in a limited range of action (8). MSC were shown to mediate T cell suppression via induction of IDO upon IFNγ activation (35, 36) and iNOS expression via Stat5 inhibition (37). As shown here, UC-MSC also retained these classical activation pathways after PHA and αCD3/CD28. However, we focused our attention to soluble mediators present in PBMNC/UC-MSC cocultures after immune activation. Similar to other reports we found a similar secretion pattern of soluble factors including GCSF, IP-10, IL6, IDO, IL-10 MIP-1a MIG, pointing to a regulatory immune signature characterized by anti-inflammatory phenotype. It has been also shown enhanced levels of IL-17 and IL-6 in the presence of MSC from adipose tissue, placenta and bone marrow (38). Further evidence has also characterized other soluble factors involved in MSC immune modulation directly targeting different cell types such as monocytes, NK cells and lymphocytes. Among key players, TNFα and IL-1β were enhanced after LPS treatment of perinatal stromal cells (placenta and amniotic fluid), identifying IL-6 secretion at early stages, as common pathway in T cell suppression (39). Similarly, IFNγ in combination with TNFα induced CXCR3 family genes (CXCL9, CXCL10, and CXCL11) and chemokines in MSC (40). Thus, it is plausible to consider soluble factors released by activated immune cells in close cooperation with UC-MSC as amplifiers of suppression processes.

Monocyte/macrophage is a critical immune cell population targeted by MSC. In co-culture studies using PBMNC-derived monocytes, MSC induced the expression of the M2-macrophage markers CD206 and CD163, promoted the reduction of IL-12 and TNFα secretion and accumulation of IL-6 and IL-10 (41). Similarly, human gingival MSC induced CD206^+^ M2-macrophages and displayed a cytokine secretion profile characterized by IL-6, CCL-2, IL-10, and GM-CSF. Furthermore, IFNγ-primed MSC also mediated monocyte reprogramming toward regulatory phenotype by pronounced secretion of IL-10 by adult MSC (42). Of note, M2-like macrophages have shown immunomodulatory and protective responses in several models (43, 44). In this study, we confirmed that activated PBMNC/UC-MSC cocultures induced the reprogramming of CD14^+^ cells toward M2-lyke phenotype, showing high expression levels of CD206, CD163 and CD301 as compared to inflammation-primed CD14^+^ cells. CD206/CD163^high^ cells also secreted higher levels of MCP-1, G-CSF, IL-6, IL-10, IP-10, and IL-4 than CD206/CD163^low^ monocytes. Importantly, induced CD206/CD163^high^ cells were able to supress proliferation of αCD3/CD28-activated T cells. Consistent with previous reports, we demonstrated that UC-MSC are also able to induce a specific immune modulatory milieu that reprograms monocytes toward M2-lyke phenotype with a subsequent induction of T cell suppression. Thus, monocytes become more tolerogenic in a cytokine microenvironment highly influenced by immune activated UC-MSC, in order to trigger immune suppression at cell and tissue level. Noteworthy, MSC derived from AMD3100/G-CSF-mobilized peripheral blood induced stronger phenotypic switch from M0 toward M2 as compare to BM derived MSC, pointing to G-CSF as critical mediator of macrophage switch (45). Here, we identified G-CSF as pivotal for M2-macrophage reprogramming. Thus, induced expression of G-CSF by immune-primed UC-MSC might be a key driver of M2-phenotype leading to a more tolerogenic macrophage enrichment, further inducing suppressive cytokine microenvironment that ultimately lead to immune remodeling. Further research should clarify the molecular mechanisms behind G-CSF-driven monocyte reprogramming by UC-MSC. Additional mechanisms of MSC-mediated immune control have strongly suggested a direct targeting of T lymphocyte differentiation and turn-over. A pivotal role of CD276/B7-H3 axis has been previously proposed for UC-MSC-mediated immunomodulation upon PHA stimulation on PBMNC (46), showing increase in naïve T cells and decrease in memory T cells associated with downregulation of IL-2, IL-12, TNF-a, and IFNγ. More recently IFNγ was identified as principal trigger of PD-L1/PD-1 axis in adipose MSC, significantly contributing to upregulation of different EXO-derived miRNAs involved in immunomodulation (47). In that regard, PD-L1/PD-1 axis has become of particular interest in the biology of MSC-mediated immune modulation, pointing to cell-to-cell contact as complementary mechanism for the inhibition of T cells activation (48). Here we found a significant increase of PD-L1 expression in UC-MSC upon immune stimulation. Given the fact that, in our experimental setting, activated UC-MSC triggered PD1/PD-L1 axis thereby inducing immunological checkpoints, it is plausible to think that immune-active UC-MSC share similar mechanisms of immune control already described in several pathways implicated in the maintenance of physiologically self-tolerance that potentially limit the duration and amplitude of immune responses. Finally, immune-activated UC-MSC subjected to an inflammatory environment not only produced large amounts of chemokines to attract lymphocytes but also enhanced the expression of adhesion molecules such as ICAM-1, HCAM, and VCAM. We believe that such upregulation modulates the homing of UC-MSC and renders UC-MSC more adhesive to T cells and monocytes, thereby enhancing UC-MSC-mediated immunosuppression. Previous reports have shown that the greater expression of ICAM-1 and VCAM-1 by MSC, the stronger immunosuppressive effects they exhibit (49, 50). Once immune cells are attached to MSC, they activate immunosuppressive signals and undergo apoptosis, cell cycle arrest or phenotype-switch (51). In these reports, strategies based on antibody blockage targeting ICAM-1 and VCAM-1, reversed the suppression of T cell proliferation in MSC/T cell cocultures when added individually or combined. Moreover, when using ICAM-1–deficient MSC isolated from ICAM-1−/− mice, impaired immunosuppressive effects *in vitro* and *in vivo* were recorded. Conversely, based on a murine model of GVHD, enforced expression of ICAM-1 on MSC led to enhanced immunosuppressive effects directly on dendritic and T cells, hence ameliorating the severity of acute GVHD (52). In line with these studies, we found active regulation of the expression of adhesion molecules in UC-MSC during immunomodulation, which could be associated to inactivation of T lymphocytes and monocyte reprogramming, via cell-cell interaction with activated UC-MSC. Further research focused on the critical roles linked to adhesion function in UC-MSC, could provide new insights to be translated into MSC-based therapy settings.

**FIGURE 8:**
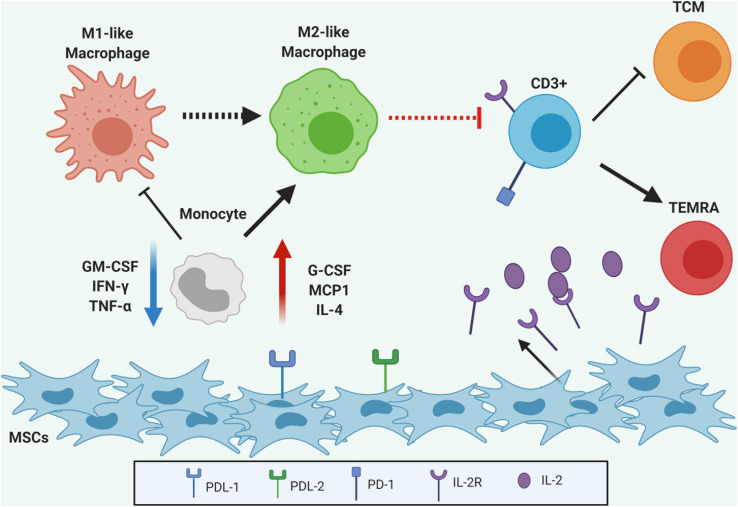
Graphical abstract. UC-MSC are able to modulate monocyte differentiation toward M2-like macrophages via secretion of multiple cytokines to the extracellular media. Additionally, a concomitant action of induced M2-like macrophages and UC-MSC secretion of IL-2R and expression of PD-L1 and PDL-2 on the extracellular membrane produce regulation of activated CD3+ T cells, reducing the pool of effector cells and inducing terminally differentiated phenotype.

Mesenchymal stromal cells have exceptional immunomodulatory properties both *in vitro* and *in vivo*, however, the complexity of involved mechanisms is not fully clarified. Here, we provided additional data on gene expression and soluble factors secretion under functional immune modulation in UC-MSC. Based on these analyses, we were able to model a variety of cell and molecular processes involved in the control of inflammation and immune activation and revealed new promising candidates in UC-MSC-mediated immune modulation (Figure 8). Mechanisms by which UC-MSC reprogram monocyte and T lymphocyte differentiation toward more tolerogenic cell types, employ extracellular cytokine secretion (i.e., G-CSF, MCP-1, IL-10, MIG, and IL-4) to favor M2-like macrophage generation. Furthermore, a concomitant action of induced M2-like macrophages, UC-MSC-derived PD-L1/2, modulation of adhesion molecules expression and IL-2R secretion, promote the regulation of activated T cells, reducing the pool of effector cells and forcing a terminally differentiated phenotype. Besides presenting a new valuable resource to strength functional research on MSC immune biology, this study has allowed the identification of novel soluble factors and cell-contact mediators by which UC-MSC coordinates immune suppressive responses. Collectively, this data will admit additional studies focused on the generation of strategies to functionally monitor immune activated UC-MSC responses in the context of MSC-based therapies. These new markers and their molecular networks might rapidly be translated to clinical scenarios to improve MSC-therapy quality and efficacy.

## Data Availability Statement

The RNA databases have been uploaded to NCBI (ID project PRJNA661127).

## Ethics Statement

All experiments and procedures involving human participants were reviewed and approved by approval number 2017EE42064, “Comité de Investigación y Etica,” Secretaría Distrital de Salud, Bogotá, Colombia. The patients/participants provided their written informed consent to participate in this study.

## Author Contributions

MC-B designed and performed cellular experiments, analyzed and interpreted data, and drafted the article. N-FZ and NL-D processed and performed analysis of transcriptome data. CM and L-XG-A performed cellular and molecular experiments. C-CG drafted the article. LC performed qRT-PCR analyses. BC provided financial support and critically reviewed the manuscript. JG contributed to experimental conception, data interpretation and analysis regarding MSC transcriptome, and provided financial support. GS conceived and designed experiments, analyzed and interpreted data, and wrote and finally approved the manuscript. All authors have seen and approved the final draft of the manuscript.

## Conflict of Interest

The authors declare that the research was conducted in the absence of any commercial or financial relationships that could be construed as a potential conflict of interest.

## Footnotes

1 ClinicalTrials.gov
