# Supplementary figures and images for: Integrated Analysis of Transcriptome and Secretome From Umbilical Cord Mesenchymal Stromal Cells Reveal New Mechanisms for the Modulation of Inflammation and Immune Activation
Source: Front Immunol. 2020 Sep 30;11:575488. doi: 10.3389/fimmu.2020.575488 (PMC7561386; doi:10.3389/fimmu.2020.575488)

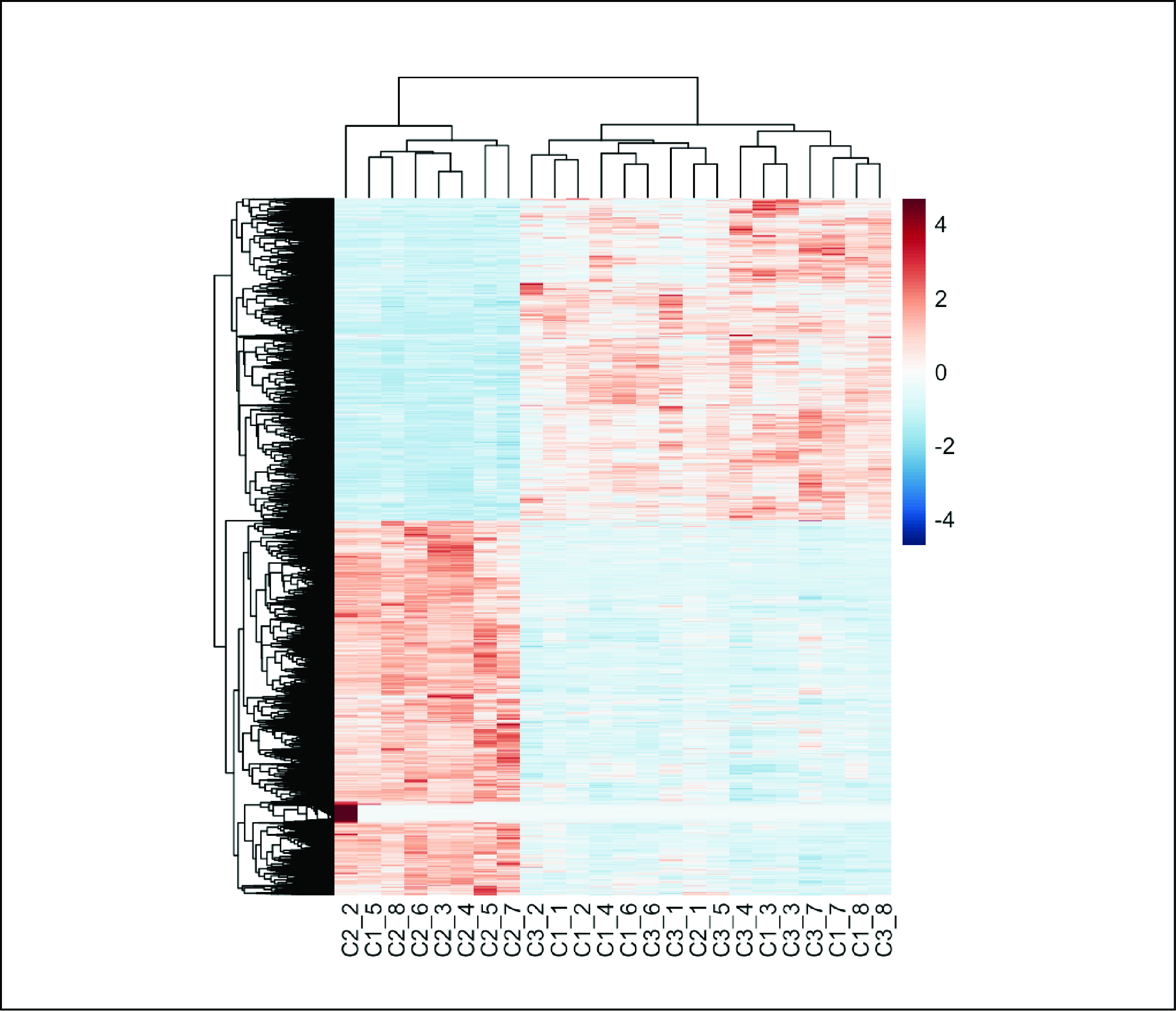

Supplement: Supplementary file 1 [file Image_1.TIF]

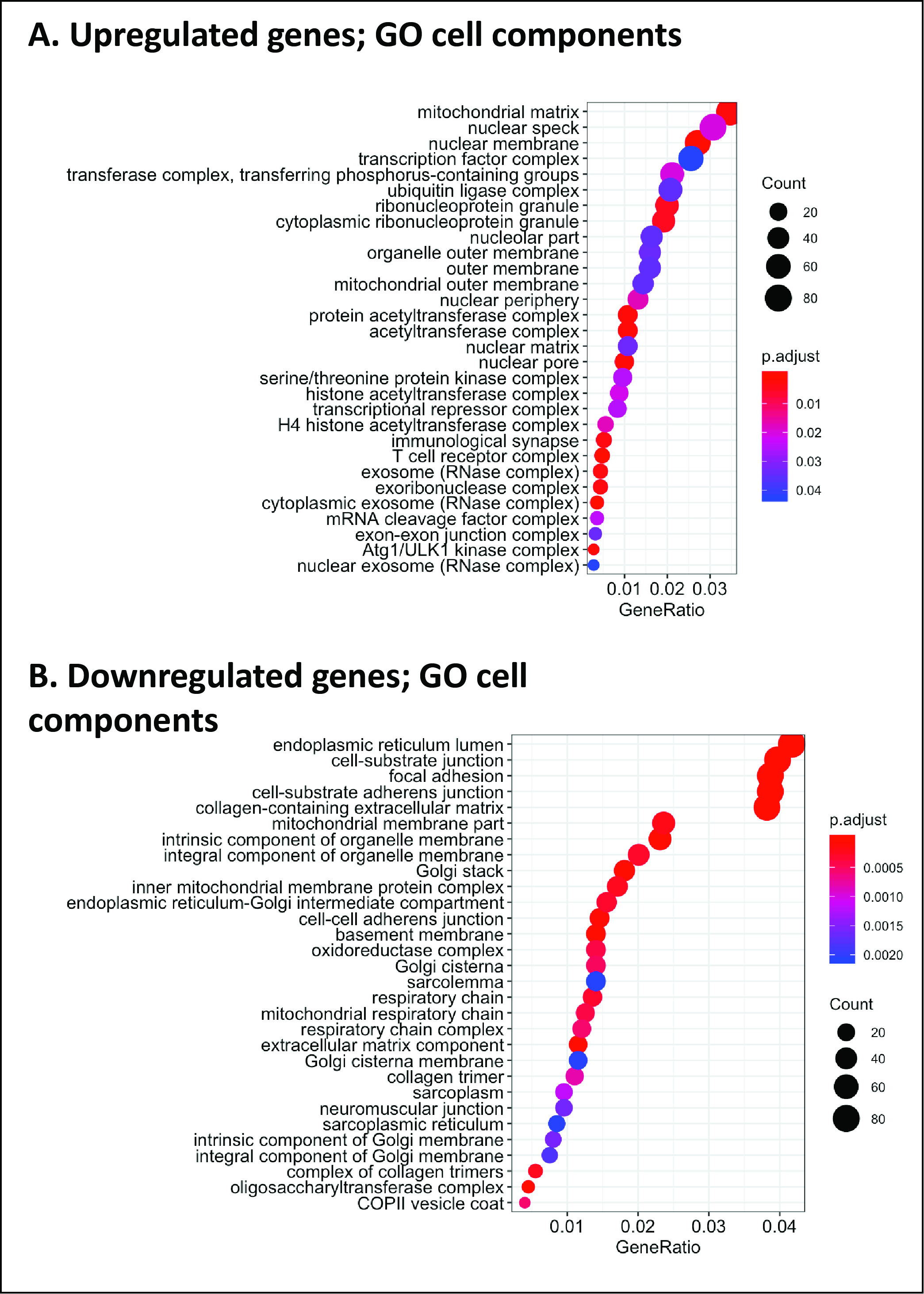

Supplement: Supplementary file 2 [file Image_2.TIF]

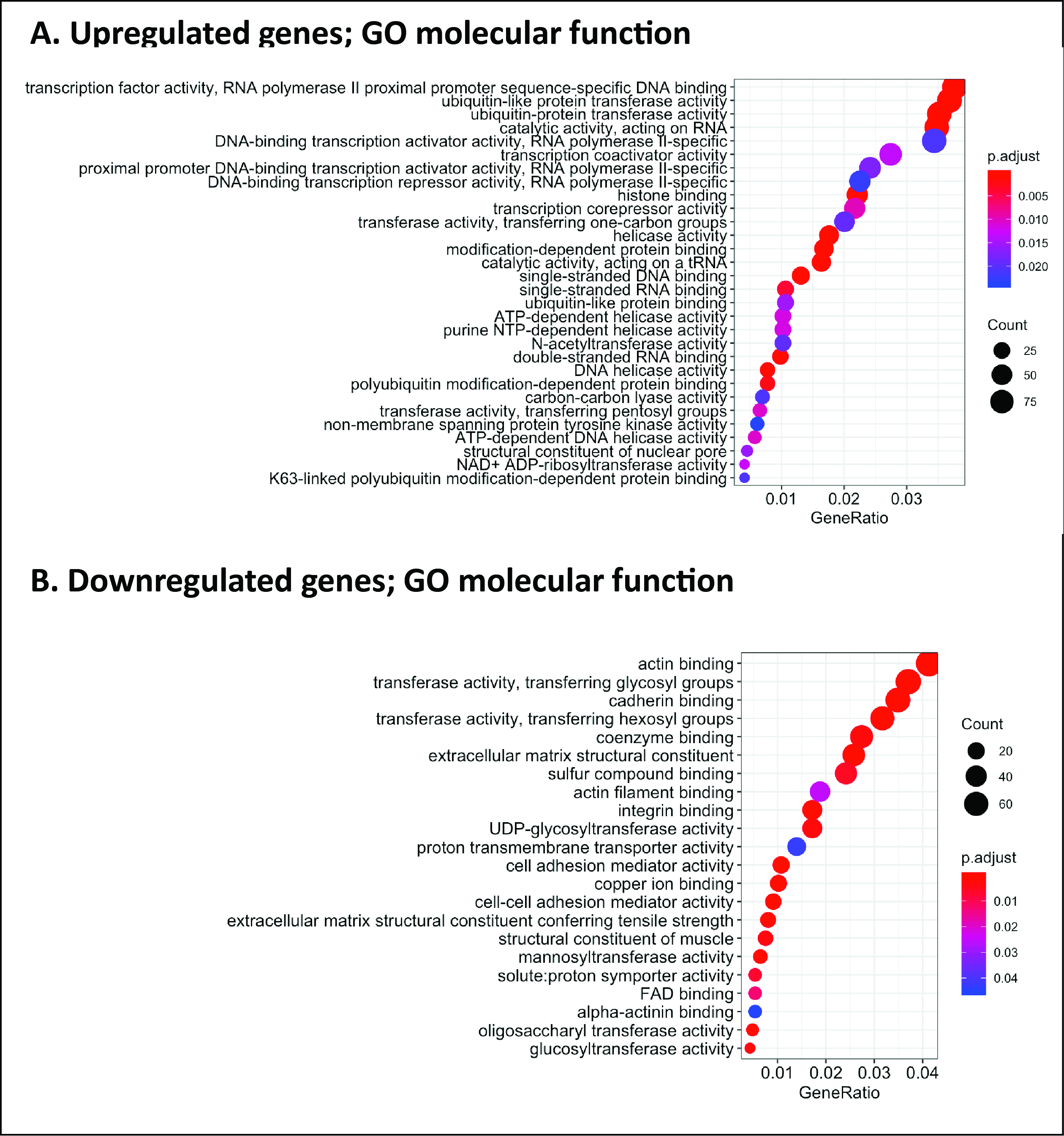

Supplement: Supplementary file 3 [file Image_3.TIF]
